# Household food insecurity and hunger status in Debre Berhan town, Central Ethiopia: Community-based cross-sectional study

**DOI:** 10.3389/fnut.2023.1035591

**Published:** 2023-03-16

**Authors:** Takele Gezahegn Demie, Getachew Tilahun Gessese

**Affiliations:** ^1^School of Public Health, St. Paul’s Hospital Millennium Medical College, Addis Ababa, Ethiopia; ^2^Department of Public Health, College of Health Science, Debre Birhan University, Debre Berhan, Amhara, Ethiopia

**Keywords:** food insecurity status, Household Food Insecurity Access Scale, hunger status category, nutrition, Debre Berhan, Central Ethiopia

## Abstract

**Background:**

Food security occurs when all people have physical, social, and economic access to sufficient, safe, and nutritious food that fits their dietary needs and food preferences for an active and healthy life at all times. There is limited evidence on this topic and not well studied in Ethiopia.

**Objective:**

This study aimed to investigate food insecurity and hunger status among households (HHs) in Debre Berhan town, Ethiopia.

**Methods:**

A community-based cross-sectional study was undertaken from 1 January 2017 to 30 January 2017. A simple random sampling technique was used to enroll 395 HHs for the study. An interviewer-administered, structured, and pretested questionnaire was used to collect data through a face-to-face interview. The household food security and hunger status were assessed by using the Household Food Insecurity Access Scale and the Household Hunger Scale, respectively. Data were entered and cleaned using Epiata 3.1 and exported to SPSS software version 20 for statistical analysis. Logistic regression was fitted, and an odds ratio with a 95% confidence interval (CI) and a value of *p* of less than 0.05 were used to identify factors associated with food insecurity.

**Results:**

A total of 377 HHs participated in the study with a response rate of 95.4%. The proportion of households with food insecurity was 32.4%, among which mild, moderate, and severe food insecurity accounted for 10.3, 18.8, and 3.2%, respectively. The mean score of the Household Food Insecurity Access Scale was 1.88 ± 3.5. Hunger occurred among 3.2% of households. The mean score of the Household Hunger Scale was 2.17 ± 1.03. Husband or male cohabitant’s occupation (AOR = 2.68; 95% CI: 1.31–5.48) and wife or female cohabitant’s literacy (AOR = 3.10; 95% CI: 1.01– 9.55) were the only factors associated with HH food insecurity.

**Conclusion:**

HH food insecurity and hunger in Debre Berhan town were unacceptably high, which can hamper achieving national targets for food security, nutrition, and health. Intensified efforts are further needed to accelerate the decline in food insecurity and hunger prevalence. Therefore, interventions need to target self-employed merchants in small businesses and women who are uneducated.

## Introduction

Food security (FS), as defined by the United Nations Food and Agriculture Organization (FAO), occurs when all people have physical, social, and economic access to sufficient, safe, and nutritious food that fits their dietary needs and food preferences for an active and healthy life at all times (1). Global development efforts target food security as a key component that needs action and are considered a stepping stone for other development goals through eradicating poverty and hunger (2, 3). Despite a Sustainable Development Goal (SDG) to eradicate hunger by 2030 (4) and international recommendations and pledges to achieve universal food security (5–7), so far, it remains a challenge to the world, and it is now becoming clear that the world cannot meet the target unless strict actions are undertaken to accelerate the progress (1, 8).

Therefore, globally, it is projected that 8% of the world’s population will still face hunger by 2030, with no improvement shown since 2015 (1). In 2021, approximately 3.1 billion people lacked access to appropriate food, and approximately 11.7% of the global population faced severe food insecurity (1). The gender disparity in the prevalence of moderate or severe food insecurity widened even further, with women experiencing 10% more moderate or severe food insecurity than men globally (8).

Food insecurity (FINS) has also been identified as a risk factor for malnutrition in studies (9, 10). It has a negative influence on people’s health and nutrition, especially those who are most vulnerable (1, 8). The prevalence of undernourishment grew from 9.3 in 2020 to 9.8 in 2021. With no progress since 2012, approximately one in three women aged 15 to 49 years (571 million) were affected by anemia in 2021 (1), globally. Moreover, stunting and wasting affect 22.0 and 6.7% of children under the age of 5 years, respectively (1, 8).

Given that food insecurity is a global concern and the prevalence of household food insecurity varies depending on a variety of factors, it is necessary to investigate the scope of the problem and the factors influencing it in various contexts. Food insecurity was found to be 4.09% in England (10), 77.2% in northern India (11), 49% in Iran (12), and 68% in Indonesia (13). In Nigeria, FINS was found to be 49.4% (14), 87.2% (15), and 90.9% (16) among rural households conducted in 2016, 2021, and 2022, respectively.

Ethiopia is not an exception to being challenged with food insecurity. More than 10% of Ethiopians and the sub-Saharan population are chronically food insecure (17, 18). According to Ethiopian Demographic and Health Survey Reports (EDHS), the prevalence of stunting, being underweight, and wasting among children in Ethiopia changed from 52, 47, and 11% to only 37, 21, and 7% progressively declining between 2000 and 2019, over a 19-year journey (19–22). The proportion of women in the reproductive age group with chronic energy deficiency changed from 30% to only 22% between 2000 and 2016 (19, 21). In addition, a systematic review showed that FINS among female-headed households was estimated to be high in Ethiopia (23). Various pocket studies in Ethiopia indicated a high prevalence of household food insecurity with varying prevalence (24–27) from place to place in the southern parts of the country and northern Ethiopia (28).

Food insecurity was affected by multiple factors that may vary from place to place. Most studies conducted around the world found that the gender of household heads (14–16, 23, 24, 29–32), age of household heads (15, 16, 24, 32–34), family size (14, 24, 27, 35), uncoupled households (24), marital status of the head of household (25, 33), educational level of women handling food (11), educational status of household head (15, 16, 26, 29, 30, 33), and economic status (14, 32, 33, 35) were determining factors of food insecurity.

Therefore, although the impact of FINS on health and nutritional status is clear, there have been few studies in the sector, and the results of those that have been undertaken are inconsistent. Furthermore, the majority of Ethiopian studies have mostly focused on measuring FINS among rural populations overlooking the urban setup. Given the sociocultural, economic, and ecological diversity of Ethiopian cities, as well as the country’s long-term high food prices, the conditionally varied urban community FINS has received little attention. Therefore, the goal of this study was to determine household food insecurity (FINS) and associated factors in Debre Berhan town, central Ethiopia. The results will inform decision-makers, programmers, health experts, and other stakeholders on food insecurity in an urban highland, as well as provide data for related public health lessons and interventions.

## Methods

### Study design, setting, and participants

A community-based cross-sectional study was conducted to assess household food insecurity status and its determinant factors among households from 1 January 2017 to 30 January 2017 in Debre Berhan town. The town is located in the North Shewa Zone of Amhara Regional State and situated in a total area of 18,081.95 hectares. It is 130 km away from Addis Ababa to the northeast. Administratively, the town is subdivided into nine kebeles (the lowest administrative unit in Ethiopia). According to the 2013 National Labor Force Survey conducted by the Ethiopian Central Statistics Agency (36), the town had an 87,204 total population, out of which 47,354 were women. The source and study population were all the households in the town. Individual respondents in the households were the study unit.

### Sample size and sampling procedure

For sample size calculation, we used the single population proportion formula by using Epi Info StatCalc considering the following assumptions: 95% confidence level (Zα/2), 37.2% proportion of households with food insecurity (25) for prevalence (p), and 5% margin of error (d), which was 359. With a 10% non-response rate, the calculated sample size was 395. However, 377 households participated in this study, and 18 of them refused to consent to their participation.

A simple random sampling technique was used to select the sample of 395 households from all the nine kebeles proportional to population size allocation, after obtaining lists of all households in the Debre Berhan town by their respective kebele of residence from health extension workers. When respondents were not available at the time of data collection, two repeated visits were made before dropping them as non-respondents.

### Study variables

Household food insecurity status was the dependent variable, while independent variables include sociodemographic and economic variables identified to affect food security status in the study area including age, sex, religion, marital status, educational status, occupation, family size, number of children, and average monthly income of the household.

### Data collection method and quality control

A structured pretested questionnaire was used to collect the required quantitative information through face-to-face interviews. It was first prepared in English, then translated to Amharic, the local survey language, by the language experts, and again back to English to check its consistency. To determine the household FINS status/level, the Household Food Insecurity Access Scale (HFIAS) developed by the Food and Nutrition Technical Assistance (FANTA) project was used (37). HFIAS is a standardized tool that can distinguish food security status in several countries across diverse sociocultural contexts.

The questionnaire is made up of nine “occurrence” or “incidence” questions (See Table 1), which reflect a usually increasing level of FINS (access), and nine “frequency-of-occurrence” questions, which are answered as a follow-up to each occurrence question to find out how frequently the situation happened. Following affirmative responses to the nine HFIAS questions, frequency questions are used to calculate the four categories of household food insecurity prevalence (HFIAP) using the formula supplied by HFIAS version 3 (37).

**Table 1:** Distribution of households based on the incidence of food insecurity conditions in Debre Berhan town, central Ethiopia (*n* = 377).

Incidence question (*N* = 377)		No	Yes
		Frequency (%)	Frequency (%)
1(a)	Concerned about not with food to eat?	294 (78.0)	83 (22.0)
2(a)	Eating food you did not desire?	282 (74.8)	95 (25.2)
3(a)	Eating monotonous foods?	306 (81.2)	71 (18.8)
4(a)	Eating foods you did not want to eat?	305 (80.9)	72 (19.1)
5(a)	Eating smaller size of meals?	337 (89.4)	40 (10.6)
6(a)	Skipping some meals in a day?	340 (90.2)	37 (9.8)
7(a)	No food to eat at all?	368 (97.6)	9 (2.4)
8(a)	Go to bed hungry?	367 (97.3)	10 (2.7)
9(a)	Not eating anything throughout the day (24 h)?	372 (98.7)	5 (1.3)

**Figure 1:**
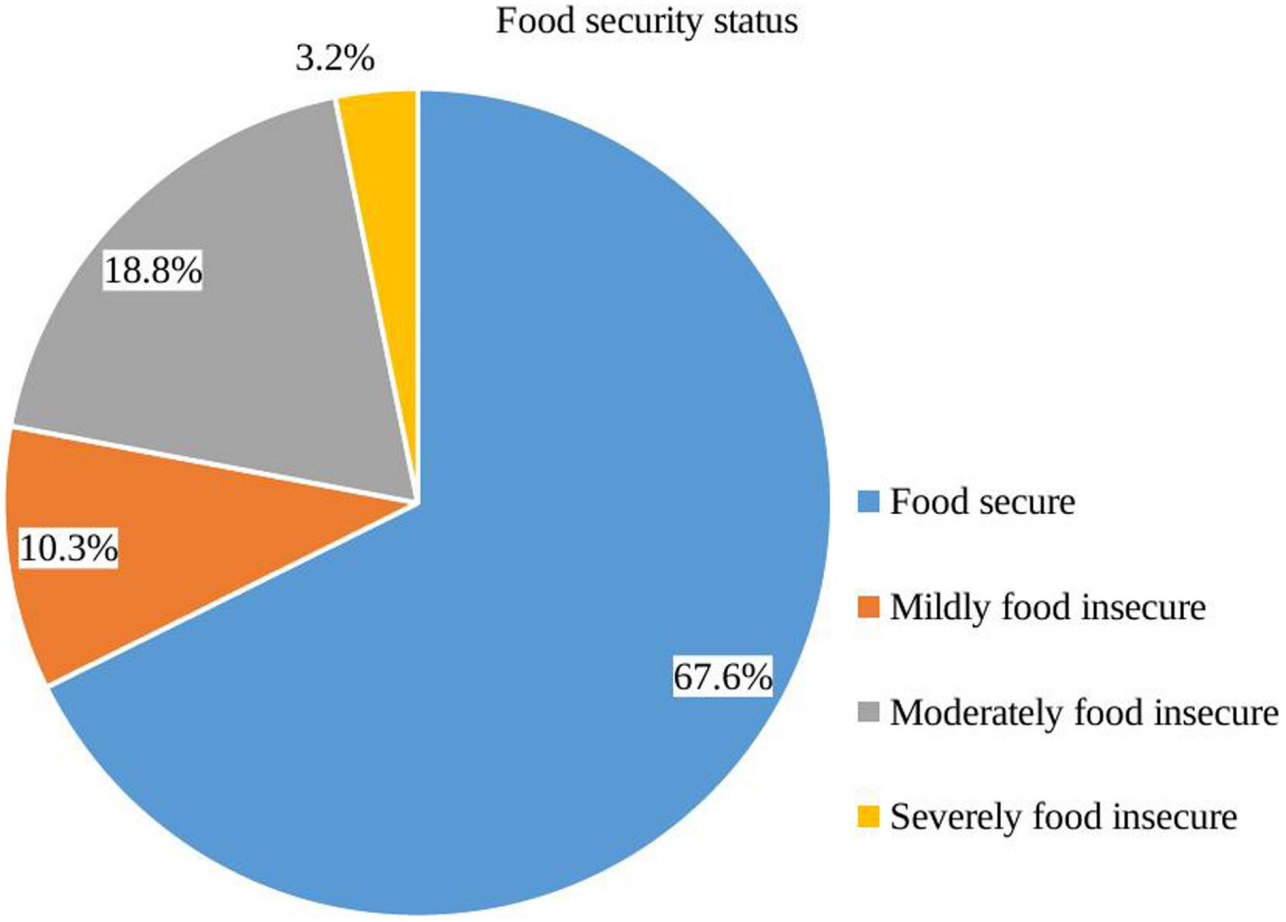
Food insecurity level of households in Debre Berhan town, central Ethiopia (*n* = 377).

According to the HFIAP indicator, there are four levels of food insecurity for households: (i) food secure, (ii) mildly food insecure, (iii) moderately food insecure, and (iv) severely food insecure (Figure 1). As households respond positively to more severe situations and/or encounter such conditions more frequently, they are classified as having a growing level of FINS. Therefore, categories were constructed according to the following criteria set in the HFIAS guideline.

A brief explanation of the categories of household food insecurity levels as assessed through the HFIAS module (37):

**Food secure (FS)**: If [(Q1a = 0 or Q1a = 1) and Q2 = 0 and Q3 = 0 and Q4 = 0 and Q5 = 0 and Q6 = 0 and Q7 = 0 and Q8 = 0 and Q9 = 0], the household did not experience any of the food insecurity situations, or only had the experience of worrying about food but rather infrequently.

**Mild food insecurity**: If [(Q1a = 2 or Q1a = 3 or Q2a = 1 or Q2a = 2 or Q2a = 3 or Q3a = 1 or Q4a = 1) and Q5 = 0 and Q6 = 0 and Q7 = 0 and Q8 = 0 and Q9 = 0], the household worries about not having food to eat occasionally or frequently, and/or being unable to consume choice foods, and/or having little variety of food, and/or some food referred to as unpalatable only on rare occasions.

**Moderate food insecurity**: If [(Q3a = 2 or Q3a = 3 or Q4a = 2 or Q4a = 3 or Q5a = 1 or Q5a = 2 or Q6a = 1 or Q6a = 2) and Q7 = 0 and Q8 = 0 and Q9 = 0], the household consumes few varieties or unpalatable foods occasionally or frequently, and/or has begun to reduce the size or number of meals infrequently or occasionally but did not experience any of the three extreme food insecurity situations.

**Severely food insecure**: If [Q5a = 3 or Q6a = 3 or Q7a = 1 or Q7a = 2 or Q7a = 3 or Q8a = 1 or Q8a = 2 or Q8a = 3 or Q9a = 1 or Q9a = 2 or Q9a = 3], the household has moved gradually to reducing the quantity of meal or number of meals most frequently, and/or experiencing the three most extreme situations such as “not having any food to eat,” “going to bed without eating any food,” or “going a whole day hungry,” even infrequently.

**Figure 2:**
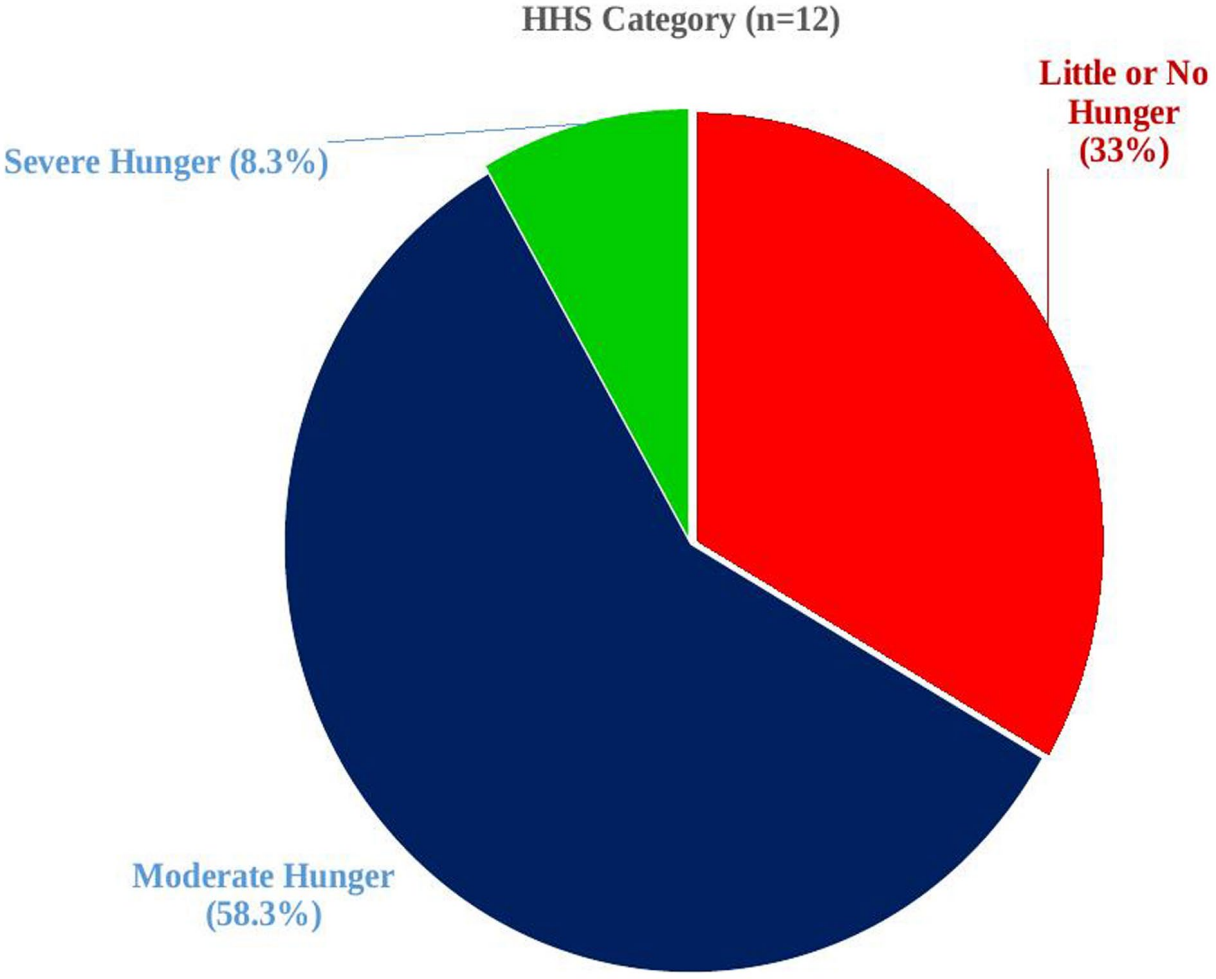
Hunger status of households in Debre Berhan town, central Ethiopia (*n* = 12).

The Household Hunger Scale (HHS) was used to assign households along a continuum of severity in food access from no hunger to severe household hunger (based on the scored value of three HHS questions) that ranges from a minimum score of 0 to a maximum score of 6. Households were categorized as no or little hunger if the score was 0–1, mild hunger if the score was 2–3, and severe hunger if the score was 4–6 (Figure 2) (38). Then, the prevalence of household food with insecurity and hunger was calculated after categorizing households into respective groups. Whereas, for the rest factors included in the study, the tool was adapted from various similar literature on the topic with some modifications to fit with the context (14, 23–25).

Six data collectors who were health professionals and had experience with household food insecurity surveys were recruited and trained to facilitate the data collection process. The training was given to data collectors for 1 day on the rationale, objective of the study, confidentiality, the process, and technique of data collection including how to use the questionnaires. A pretest was performed before the actual data collection on 5% of the sample out of the study area, and necessary modifications were made accordingly. The researchers closely supervised the data collection process on daily basis, and feedback and correction were given for incompleteness and inconsistencies throughout the data collection period.

### Data processing and analysis

The completeness and consistency of responses were further reviewed on all returned copies of the questionnaire. The data were then entered into EpiData version 3.1 for cleaning and coding, with double-entry verification, and exported to SPSS version 20 for further analysis. The descriptive statistics of the data were summarized using the mean, standard deviation, frequencies, and percentages. The dependent variable was binary coded with a “1” for food-insecure households and a “0” for food-secure households to compute binary logistic regression. To identify factors independently related to household food insecurity, a bivariate logistic regression analysis was used to identify the relationships between the dependent and independent variables at a value of *p* of 0.2 cutoff point to include in the multivariable logistic regression model. The fitness of the multivariable regression model was tested through the Hosmer–Lemeshow goodness-of-fit. The adjusted odds ratio (AOR) with 95% of confidence intervals at a value of *p* of ≤0.05 was used to declare the variables significantly associated with the dependent variable in the multivariable analysis. Finally, data were presented using tables, figures, and text descriptions.

### Ethical considerations

The Institutional Ethical Review Board (IRB) of Debre Berhan University granted ethical approval. Support letters were obtained from the research, community service, and Postgraduate Coordination Office of the College of Health Sciences as well as from the Debre Berhan town’s health office. Because some respondents were unable to read or write, each participant gave their informed verbal agreement after being told of the study’s aim, advantages, risks, information confidentiality, and voluntary nature of participation. Furthermore, all data collectors and investigators ensured the confidentiality of the information gathered from each study subject by using code numbers instead of personal identifiers and making the questionnaire inaccessible to anyone other than the investigators.

## Results

### Sociodemographic characteristics

A total of 377 HHs participated in this study, making a response rate of 95.4%. The sociodemographic characteristics of the study unit are presented in Table 2. The vast majority of HHs were male-headed (88.4%) and more than half of the heads of HH were below 30 years of age (56.1%). The majority of respondents were Orthodox Christian by religion (89.3%), married (94.5%), and the husband or male cohabitant partner’s occupation was a governmental employee (61%). Approximately 89% of couples or partners attended formal education. More than half (60%) of the HHs had families of four to six members, with an average of one child under the age of five in HH (311, 85.2%), a maximum of two children per HH (290, 77.6%), and an income of at least the group’s median monthly earnings (216, 57.3%). The household’s monthly income ranges from 100 to 20,000 Ethiopian Birr. In the U.S. dollar equivalent, these values become approximately 4.3 to 869.6 using the currency exchange rate since April 2017 [1 USD is nearly 23 ETB at that time].

**Table 2:** Sociodemographic characteristics of respondents and households in Debre Berhan town, central Ethiopia (*n* = 377).

Characteristics	Response categories	Frequency	Percent
Age of household head (*n* = 374)	19–29	210	56.1
	30–39	152	40.6
	> = 40	12	3.2
Sex of household head (*n* = 371)	Father headed	328	88.4
	Mother headed	43	11.6
Religion of household head (*n* = 374)	Orthodox	334	89.3
	Protestant	26	7.0
	Others	14	3.7
Marital status household head (*n* = 365)	Married	345	94.5
	Others^1^	20	5.5
Occupation of husband/cohabitant male partner (*n* = 344)	Daily laborer	37	10.8
	Merchant	63	18.3
	Private sector employee	34	9.9
	Governmental employee	210	61.0
Education of husband/cohabitant male partner (*n* = 364)	Uneducated	18	5.0
	Informal education	22	6.0
	Formal education	364	89.0
Educational status of wife/cohabitant female partner (*n* = 375)	Uneducated	19	5.1
	Informal education	20	5.3
	Formal education	336	89.6
Occupation of the wife/cohabitant female partner (*n* = 370)	Housewife	141	38.1
	Merchant	57	15.4
	Private sector employee	34	9.2
	Governmental employee	138	37.3
Family size (*n* = 296)	<=3	112	37.8
	4–6	176	59.5
	> = 7	8	2.7
Number of children (*n* = 374)	1	167	44.7
	2	123	32.9
	> = 3	84	22.4
Number of under 5 children (365)	1	311	85.2
	> = 2	54	14.8
Household monthly income (*n* = 377)	Below median	161	42.7
	Median and above	216	57.3

1 Separated, divorced, widowed, cohabitant partner.

### Food security status of households

The HFIAS module of nine occurrence/incidence questions for FINS among households in the study region is shown in Table 1. It indicated 78, 74.8, 81.2, and 80.9% of household questions responded “no” to the occurrence questions 1–4, while 89.4, 90.2, 97.6, 97.3, and 98.7% of them responded “no” to the occurrence questions 5–9. The remaining households answered the nine HFIAS questions positively (“yes”). Table 1 also shows a decreased trend in the percentage of households that replied affirmatively to the nine HFIAS questions with a 4-week recall interval, while there is a steady increase in the percentage of households that responded “no” to the questions.

**Figure 3:**
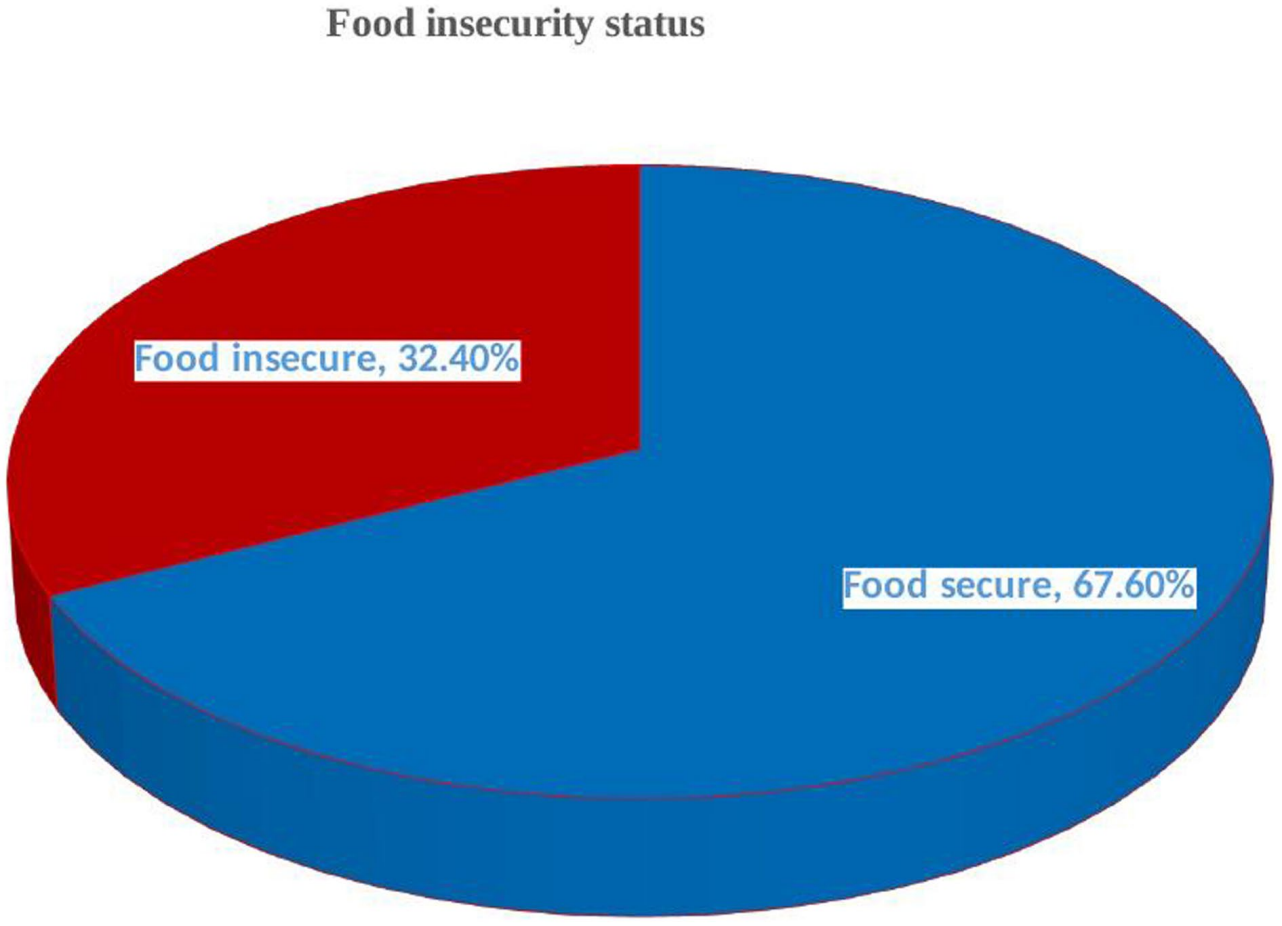
Food security status (secure vs. insecure) in Debre Berhan town, central Ethiopia (*n* = 377).

Approximately 32.4% of households were food insecure (Figure 3) with approximately 10, 19, and 3% facing mild, moderate, and severe FINS (Figure 1), respectively. The mean Household Food Insecurity Access Scale (HFIAS) score was 1.88 ± 3.5 (standard deviation). The mean and standard deviation Household Hunger Scale (HSS) score was 2.17 ± 1.03. Among households with hunger (3.2%), more than half of them were in a stage of moderate hunger (58.3%) while those in the severe hunger stage accounted for 8.3% (Figure 2).

### Factors associated with food insecurity

Bivariate logistic regression revealed multiple factors associated with food insecurity. In the multivariable analysis, after we adjusted for confounding variables, only the occupation of the husband or male cohabitant and the educational status of the wife or female cohabitant were found to be the determining factors of food insecurity among HHs in Debre Berhan town. The odds of HH food insecurity were approximately two times higher (AOR = 2.68; 95% CI: 1.31–5.48) among husbands or male cohabitants who are merchants than government employees. In addition, the odds of HH food insecurity were approximately three times higher (AOR = 3.10; 95% CI: 1.01–9.55) among uneducated wives or female cohabitants compared with women with formal education (Table 3).

**Table 3:** Factors associated with food insecurity using multivariable logistic regression among households in Debre Berhan town, central Ethiopia (*n* = 377).

Variables	Food insecure				
	Percent (%)	COR	95% CI	AOR	95% CI
Age of household head					
19–29 (*n* = 210)	33.3	0.35	0.11–1.17	0.32	0.07–1.52
30–39 (*n* = 152)	28.9	0.29	0.09–0.97	0.25	0.05–1.21
≥40 (*n* = 12)	58.3	1		1	
Sex of household head					
Father headed (*n* = 328)	30.5	1		1	
Mother headed (*n* = 43)	46.5	1.98	1.04–3.77	1.37	0.53–3.51
Religion of household head					
Orthodox (*n* = 334)	33.5	1		1	
Protestant (*n* = 26)	15.4	0.36	0.12–1.07	0.34	0.09–1.30
Others (*n* = 14)^1^	37.7	1.10	0.36-3.36	0.70	0.19–2.60
Occupation of husband/cohabitant male partner					
Daily laborer (*n* = 37)	27.0	1.10	0.50–2.42	1.77	0.68–1.62
Merchant (*n* = 63)	42.9	2.22	1.23–4.00	**2.68**	**1.31–5.48** *
Private sector employee (*n* = 34)	41.2	2.07	0.98–4.39	2.23	0.96–5.20
Governmental employee (*n* = 210)	25.2	1		1	
Education of husband/cohabitant male partner					
Uneducated (*n* = 18)	66.7	4.68	1.71–12.83	1.82	0.49–6.72
Informal education (*n* = 22)	22.7	0.69	0.25–1.92	1.09	0.27–4.44
Formal education (*n* = 324)	29.9	1		1	
Education of wives/cohabitant female partner					
Uneducated (*n* = 19)	63.2	3.77	1.44–9.85	**3.10**	**1.01–9.55** *
Informal education (*n* = 20)	20.0	0.55	0.18–1.69	0.31	0.07–1.46
Formal education (*n* = 336)	31.3	1		1	
Occupation of wives/cohabitant female partner					
Housewife (*n* = 141)	27.0	0.84	0.50–1.42	0.55	0.28–1.08
Merchant (*n* = 57)	43.9	1.79	0.95–3.38	1.19	0.53–2.66
Private sector employee (*n* = 34)	47.1	2.03	0.95–4.37	1.60	0.65–3.94
Governmental employee (*n* = 138)	30.4	1		1	
Number of children					
One (*n* = 167)	37.7	1		1	
Two (*n* = 123)	28.5	0.66	0.40–1.08	2.28	0.94–5.50
> = Three (*n* = 84)	28.6	0.66	0.37–1.17	1.59	0.68–3.67

* Likelihood-ratio test, a value of *p* of < 0.05.

1 Muslim and Catholic.

COR, crude odds ratio; AOR, adjusted odds ratio; CI, confidence interval.

## Discussion

Food insecurity is a major challenge in Ethiopian HHs. This study investigated the level and determinants of HH food insecurity using HFIAS in Debre Berhan town (urban area). A total of 122 (32.4%) HHs had FINS (10.3, 18.8, and 3.2% were mildly, moderately, and severely food insecure, respectively). The overall hunger prevalence in the study subjects was 3.2%; among them, 33.3, 58.3, and 8.3% had little, moderate, and severe hunger, respectively. Merchant husbands’ or male cohabitant partners’ occupation and educational status of wives or female cohabitant partners were both found to be significant predictors of HH food insecurity in the research area.

The FINS revealed (32.4%) by this study is lower compared with the findings of different studies in northern India (77.2%), Iran (49%), Indonesia (68%), Nigeria (49.4%), Sidama, Ethiopia (82.3%), southwest Ethiopia (42.9%), Abaya, Ethiopia (38.1%), Wolaita Sodo, Ethiopia (37.6%) (11–15, 23–27). However, HH food insecurity was higher than the prevalence in England (4.09%) and Wollo, Ethiopia (20.9%) (10, 28). In all cases, the discrepancies could emanate from the time factor, methodology, and differences in socioeconomic status or infrastructure. The coincidence of the data collection with a harvest season where food is more plentiful and costs are relatively low could be a cause for the variance. As a result, lesser HH food insecurity in this study could be linked to the harvest season of the year in which the survey was conducted in addition to study setting differences. Moreover, most of the other studies included rural HHs as well, while our study is conducted in an urban context. On the other hand, the prevalence was higher than some of the findings stated which could be the result of the study setting and socioeconomic disparities. Nevertheless, the prevalence of FINS and hunger in the area was unacceptably high as seen in the light of the SDGs indicator (4), which targets zero hunger and assures FINS for all nations before 2030. In addition, the FINS in the area hampers the progress toward the national target set in the Health Sector Transformation Plan (HSTP) II (39) of the country to tackle malnutrition and prevention and control of communicable and noncommunicable diseases as well.

HH food insecurity was found to be associated with the occupation of a husband or male cohabitant partner. The odds of FINS were 2.68 times higher among HHs with husbands or male cohabitant partners being merchants than government employees. This could be since most merchants in the Ethiopian context are engaged in small-scale private businesses that may not sufficiently cover the monthly family expense for food and allied basic needs. Studies revealed that HH food insecurity in different parts of the world is associated with lower income, the head of the HH is engaged in low-level jobs such as daily laborers, lower monthly expenditure for food, and overall economic status (14, 25, 33–35).

HH food insecurity was also determined by the educational status of wives or female cohabitant partners in the HH. Moreover, the odds of FINS were approximately three times higher among HHs with uneducated wives or female cohabitant partners compared with those with formal education. This finding goes in line with the findings from other similar studies stating that the educational status of women handling the food in the household (11) and the head of the HH (28, 30, 31, 35) determines FINS. It could be due to lower educational status also affecting monthly earnings and awareness level of the importance of expenditure of increased share of the total income for food to improve health and wellbeing of the family members among others, and thereby increased productivity and income in return. It may also be related to the lower awareness level leading to lower family planning service utilization, thereby leading to larger family sizes and consequently becoming food insecure.

This study is not without limitations. There could be a possibility of recall bias since HFIAS relies on the recall of events that occurred within the last 4 weeks. Because the study period coincided with the harvest season, HH food insecurity in the setting may have been minimally underestimated, even though seasons may not affect urban FINS like that of the local rural farming communities. Aside from that, the study’s potential weakness is concerned with HH income, which is estimated only using an oral report of respondents than a wealth index.

## Conclusion

Household (HH) food insecurity and hunger in the study area were unacceptably higher and hamper achieving the global and national targets for food security, nutrition, and health. The occupation of the husband or male cohabitant partner and the educational status of the wife or female cohabitant partner in the HH determined household food insecurity. Despite the progress in improving the food security status of the country so far, intensified efforts are further needed to accelerate the decline in the prevalence of food insecurity and hunger in the area. Therefore, interventions need to target self-employed merchants in small businesses and women who are uneducated.

## Data availability statement

The original contributions presented in the study are included in the article/supplementary material, further inquiries can be directed to the corresponding author.

## Ethics statement

The studies involving human participants were reviewed and approved by the Institutional Review Board of Debre Berhan University. Written informed consent for participation was not required for this study in accordance with the national legislation and the institutional requirements.

## Author contributions

TD contributed to the conception and design and data collection of the study, wrote the sections of the manuscript, conducted the research, and had primary responsibility for the final content. TD and GG supervised, organized the database, and performed the statistical analysis. GG wrote the first draft of the manuscript. All authors contributed to manuscript revision, read, and approved the submitted version.

## Funding

Debre Berhan University sponsored the fieldwork of this research. The supporting organization had no role in the study design; collection, analysis, and interpretation of data; writing of the report; and no restrictions regarding publication.

## Conflict of interest

The authors declare that the research was conducted in the absence of any commercial or financial relationships that could be construed as a potential conflict of interest.

## Publisher’s note

All claims expressed in this article are solely those of the authors and do not necessarily represent those of their affiliated organizations, or those of the publisher, the editors and the reviewers. Any product that may be evaluated in this article, or claim that may be made by its manufacturer, is not guaranteed or endorsed by the publisher.

## Abbreviations

AOR: adjusted odds ratio
CI: confidence interval
COR: crude odds ratio
EDHS: Ethiopian Demographic and Health Survey Reports
FANTA: Food and Nutrition Technical Assistance
FAO: Food and Agriculture Organization
FINS: food insecurity
FS: food secure
HFIAS: Household Food Insecurity Access Scale
HHs: Household(s)
HFIAP: Household Food Insecurity Prevalence
HHS: Household Hunger Scale
HSTP: Health Sector Transformation Plan
IRB: Ethical Review Board
SDG: Sustainable Development Goal.
